# Solitary nasal trichoepithelioma

**DOI:** 10.1016/S1808-8694(15)30618-2

**Published:** 2015-10-18

**Authors:** Lucas Gomes Patrocinio, Priscila Garcia Damasceno, Tomas Gomes Patrocinio, José Antonio Patrocinio

**Affiliations:** 1 Otorhinolaryngologist, physician of the Otorhinolaryngology Unit, Medical School of the Universidade Federal de Uberlandia.; 2 Physician, resident of the Otorhinolaryngology Unit, Medical School of the Universidade Federal de Uberlandia.; 3 Physician, resident of the Otorhinolaryngology Unit, Medical School of the Universidade Federal de Uberlandia.; 4 Professor Titular, Chefe of the Otorhinolaryngology Unit, Medical School of the Universidade Federal de Uberlandia. Otorhinolaryngology Unit, Medical School of the Universidade Federal de Uberlandia, Minas Gerais, Brasil.

**Keywords:** plastic surgery, skin neoplasms, nasal neoplasms

## INTRODUCTION

Trichoepitheliomas are benign cutaneous neoplasms occurring mostly on the face, which derive from pillous follicles.^1^ There are two clinical presentations: a hereditary sex-linked multiple form that usually affects the face, scalp and upper thorax of young adults; and a non-hereditary solitary form that may affect any part of the body (but mostly the face) of adults.^1^ The diameter is rarely more than 2 to 3 cm.2 It is an extremely rare tumor; in the literature we surveyed, there were only two cases of trichoepitheliomas involving the skin in the nose area.^3^, ^4^

This paper reports a case of a solitary nasal trichoepithelioma that was removed surgically, followed by a flap rotation. We discuss the diagnostic and therapeutic aspects of this rare skin tumor.

## CASE REPORT

**Figure 1:**
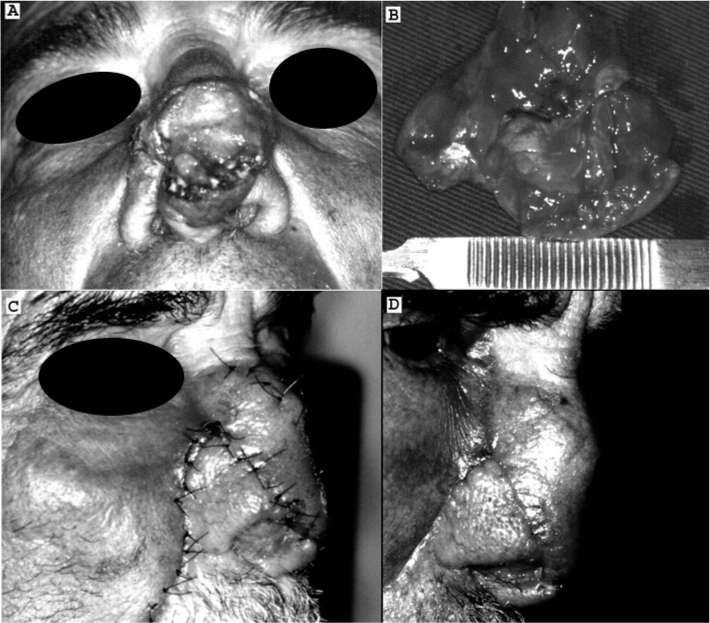
Pictures showing a solitary nasal trichoepithelioma (A) removed surgically (B) and after reconstruction with a nasogenian flap (C e D).

JCM, a male patient aged 56 years, complained of a slow-growing lesion on the dorsum of the nose during the past five years. The physical examination showed a brownish nodule on the dorsum of the nose, with visible blood vessels, a fibroelastic consistency, and measuring 3.0 x 2.5 cm (Figure 1A). The initial diagnosis was a basocellular carcinoma. The nodule was resected surgically (Figure 1B) and sent to the pathology department, which described islands of basaloid cells within small cystic structures filled with laminated keratin and epithelial cells to form invaginations similar to follicular papillae. A diagnosis of trichoepithelioma was confirmed and the tumor was shown to have free margins. A nasogenian flap was used for reconstructing the nose (Figures 1C and 1D). The patient was monitored for 48 months with no recurrences.

## DISCUSSION

Solitary trichoepitheliomas are non-hereditary benign trichogenic tumors of very low incidence that affect adults.^2^ We found only two cases of solitary nasal trichoepitheliomas in the literature.^3^, ^4^

The multiple and solitary forms are morphologically identical; they may be differentiated by the number of lesions^2^, ^3^ These tumors are firm, translucent nodules or papules.^5^

The differential diagnosis of the solitary form is made mostly with the basocellular carcinoma, but also with the trichofoliculoma, melanocytic nevi and sebaceous hyperplasia. Histopathology provides the final diagnosis.^1^

Histologically, it is a well-differentiated tumor containing hair-forming structures and keratinous cysts surrounded by basophilic cells in an adenoid pattern.^5^

Generally, surgical removal of the lesion for pathology is curative. In 1999, Jemec et al.^5^ reviewed the first ten cases of the literature and found a recurrence in only one case after surgical removal.

## FINAL COMMENTS

Solitary trichoepitheliomas are extremely rare benign tumors that should be considered when finding a single solid nodule or papula on the face. Confirmation by excision biopsy is essential for the diagnosis and treatment.
